# Surge of Miller Fisher variant and Guillain‐Barré syndrome in two downtown Los Angeles community teaching hospitals

**DOI:** 10.1002/ccr3.3132

**Published:** 2020-07-16

**Authors:** Michael C. Yang, Antonio Liu

**Affiliations:** ^1^ Department of Internal Medicine Adventist Health White Memorial Los Angeles CA USA; ^2^ Department of Neurology Adventist Health White Memorial Los Angeles CA USA; ^3^ Department of Neurology California Hospital Medical Center Los Angeles CA USA

**Keywords:** ganglioside antibody tests, Guillain‐Barré syndrome, Los Angeles, Miller Fisher syndrome, Miller Fisher variant, ophthalmoplegia

## Abstract

Guillain‐Barré syndrome (GBS) and Miller Fisher variant (MFv) cases spiked threefold in Los Angeles, with a high proportion of MFv cases. MFv is underdiagnosed when accompanying neurological symptoms are mild. This report emphasizes the seasonality of GBS and its relation to ganglioside antibodies.

## Graphical abstract

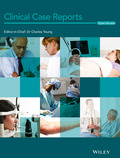

## 1 INTRODUCTION

Guillain‐Barré syndrome (GBS) is a group of acute polyneuropathies that occurs after an antecedent illness that triggers antibodies that interfere with nerve function. Miller Fisher variant (MFv) is a subtype of GBS. We report a threefold surge of MFv and GBS cases in two downtown Los Angeles community teaching hospitals.

In 1916, Guillain, Barré, and Strohl first described Guillain‐Barré syndrome (GBS) as an acute flaccid paralysis in which patients lacked deep tendon reflexes (DTRs) and possessed albuminocytological dissociation in the cerebrospinal fluid (CSF).^1^ In the 1950s, Miller Fisher noted a variant of GBS in which patients presented with a triad of ophthalmoplegia, ataxia, and areflexia.^2^ GBS has an incidence of 1‐2 cases per 100 000 person‐years worldwide and increases with age—after 10 years old, there is a 20% increase in incidence with every decade of life.^3^, ^4^ Miller Fisher variant (MFv) has an incidence of 1‐2 cases per 1 000 000 person‐years.^5^ MFv is extremely rare and only occurs in approximately 5% of GBS cases in the United States.^1^ However, in Japan, MFv is reported to be present in over 25% of GBS cases.^6^ Clearly, there are some environmental, geographic, and possible genetic factors that may affect the incidence of GBS and its variants.

Guillain‐Barré syndrome (GBS) is typically preceded by an inciting respiratory or gastrointestinal infection. Oftentimes, these illnesses are mild and underreported by patients. Over 90% of GBS cases are associated with an infection, and the usual suspects include *Campylobacter jejuni, Mycoplasma pneumonia, Haemophilus influenza*, cytomegalovirus (CMV), Epstein‐Barr virus (EBV), and Enterovirus infections.^5^ Other reported triggers of GBS include pregnancy, psychological stress, and H1N1 influenza vaccination.^3^, ^5^, ^7^ MFv, like GBS and other acute autoimmune neuropathies, occurs when antibodies initially produced to target a pathogenic invader cross‐reacts with structurally similar self‐proteins.^8^ In the case of GBS and its variants, the proteins of peripheral nerves are damaged in the immunological crossfire resulting in varying patterns of neuropathy.^6^, ^8^

Diagnosis of GBS and MFv is based on clinical findings, but can be supported by cerebrospinal fluid (CSF) analysis, electrodiagnostic studies, and ganglioside antibody testing. Commonly tested antibodies include, but are not limited to, GM1, GM1b, GD1a, GD1b, GT1a, GT1b, and GQ1a.^9^ The particular antecedent infectious microorganism has been known to be associated with specific ganglioside antibodies—each with their own mechanism of action.^10^ Given its immunological basis, GBS and its variants are treated with intravenous immunoglobulin (IVIG) and plasma exchange.^11^ Both treatments are equally effective, and decision is based on availability and side effect tolerance.^11^

## 2 MATERIALS AND METHODS

Downtown Los Angeles (DTLA) is served by four community hospitals—two of which are Adventist Health White Memorial (AHWM) and California Hospital Medical Center (CHMC). AHWM is a 300 bed hospital, while CHMC is a class 2 trauma center with 310 beds. Both are community teaching hospitals with Joint Commission certified Primary Stroke Centers. The inpatient neurologic consultation services are provided by the same neurology medical group, thus giving the authors unique firsthand observation and diagnostic experience in all cases recorded. In the years before and after 2015, each hospital will see an average of four cases of GBS per year. In the middle of 2015, the authors had recognized a surge in the number of cases in GBS and MFv. Special efforts were initiated to include and record all cases. After 2015, the authors performed a retrospective chart review based on discharge diagnosis. A second round chart review did not capture any new cases that the authors did not already have on file. After the proper consents and IRB approval were obtained, patient data were conglomerated and analyzed. Testing of Asialo‐GM1, GM1, GM2, GD1a, GD1b, and GQ1b antibodies was performed by ARUP laboratories using semiquantitative enzyme‐linked immunosorbent assays.

## 3 RESULTS

### 3.1 Clinical profiles

In 2015, a total of 17 patients were diagnosed with GBS and MFv in Adventist Health White Memorial (AHWM) and California Hospital Medical Center (CHMC): eight patients with MFv and nine patients with GBS. 47% of cases were MFv, and 53% of cases were classic GBS. Ages ranged from 23 to 67 years with the average age being approximately 46 years for the total patient group and the individual GBS and MFv groups. One patient in both of the GBS and MFv groups had reported receiving the seasonal flu shot. Only three patients (all within the GBS group) had reported a preceding illness (33%)—notably none of the patients in the MFv group recalled a preceding illness. 13/17 patients (76%) received IVIG, and 6/17 patients received plasma exchange therapy (35%). 3/17 patients received combination treatment of IVIG and plasma exchange (17%). 1/17 patients did not undergo any treatment (5%); notably, the patient was pregnant at the time of diagnosis. 3/17 patients required mechanical ventilation (17%), all of whom were in the GBS group.

### 3.2 Seasonality and timing

A majority of the documented GBS cases occurred during winter and spring (January to June, 78%), and all GBS cases occurred between February and July (Table 1). Similarly, a majority of MFv cases occurred during winter and spring (January to June, 75%). However, MFv cases were also seen in September and December (Table 2). The range of GBS cases occurred over five months, whereas the range of MFv cases occurred over 12 months. Seasons are divided into three month segments (winter: January–March, spring: April–June, summer: July– September, autumn: October–December).

**Table 1:** Classic Guillain‐Barré Syndrome (GBS) cases

Patient‐age/sex	Month	Antecedent illness	Recent vaccination	Antibodies	Clinical features	Mech vent	Treatment
1‐48/M	Feb	Flu	No	GD1a: 132 GD1b: 109 GQ1b: 238	Ascending Weakness, Mild Dysphagia, Mild Dysarthria, No Respiratory Difficulty EOM normal	No	IVIG
2‐60/M	Mar	Unknown	No	AsialoGM1: 322 GD1b: 306	Ascending Weakness, No Dysphagia, No Dysarthria, No Respiratory Difficulty EOM normal	No	IVIG
3‐53/M	Mar	No	No	GM1: 53 GQ1b: 60	Ascending Weakness, Bulbar Weakness, Respiratory Failure Some Ophthalmoplegia	Yes	Plasma Exchange, then IVIG
4‐49/M	Apr	Diarrhea	No	GD1a: 529 GD1b: 168	Ascending Weakness, No Dysphagia, No Dysarthria, No Respiratory Difficulty EOM normal	No	IVIG
5‐24/M	Apr	Diarrhea & URI	Flu shot 6 wk prior	GD1a: 127	Ascending Weakness, No Dysphagia, EOM normal	No	IVIG
6‐45/M	May	No	No	AsialoGM1: 496, GM1: 468, GD1b: 803	Ascending Weakness, Bulbar Weakness, Respiratory Failure, Some ophthalmoplegia	Yes	Plasma Exchange, then IVIG
7‐65/M	Jun	No	No	GM1: 93 GD1b: 164	Ascending Weakness, Mild Dysphagia, Mild Dysarthria, No Respiratory Difficulty, EOM normal	No	Plasma Exchange
8‐45/F	Jul	No	No	GM1: 67 GM2: 62	Ascending Weakness, No Dysphagia, No Dysarthria, No Respiratory Difficulty, EOM normal	No	IVIG
9‐32/F	Jul	No	No	GM2: 65	Ascending Weakness, Bulbar Weakness, Respiratory Failure, Ophthalmoplegia	Yes	Plasma Exchange, then IVIG

Note

Summary of GBS cases seen at Adventist Health White Memorial (AHWM) and California Hospital Medical Center (CHMC) in 2015. Details of each case are detailed, including month of illness, presence of antecedent illness, recent vaccinations, ganglioside antibody panel, clinical features of illness, need for mechanical ventilation, and treatment received.

**Table 2:** Miller Fisher Variant (MFv) cases

Patient‐age/sex	Month	Antecedent illness	Recent vaccination	Antibodies	Clinical features	Mech vent	Treatment
10‐67/F	Jan	No	No	GD1b: 146 GQ1b: 478	Ophthalmoplegia, No Limb weakness, Mild Dysphagia, Mild Dysarthria, No Respiratory Difficulty	No	IVIG
11‐29/F	Feb	No	No	AsialoGM1: 63	Pregnant, Mild Ophthalmoplegia, No Limb Weakness, No Dysphagia, Mild Dysarthria, No Respiratory Difficulty	No	No treatment
12‐59/M	Mar	No	No	GD1b: 373	Ophthalmoplegia, Dysphagia, Dysarthria No Respiratory Difficulty, Mild Limb Weakness	No	IVIG
13‐41/M	Apr	No	No	GQ1b: 496	Ophthalmoplegia, No Dysphagia, No Dysarthria, Lower Extremity Weakness, Relative Sparing of Upper Extremity, a GBS‐MFv Overlap	No	Plasma Exchange
14‐23/F	May	No	Yes	GQ1b: 51	Mild Ophthalmoplegia, No Limb Weakness, No Dysphagia, No Dysarthria, No Respiratory Difficulty	No	IVIG
15‐53/M	Jun	No	No	GQ1b: 68	Ophthalmoplegia, Mod Bulbar Weakness Mild Limb Weakness Mild Ataxia, A GBS‐MFv Overlap	No	IVIG
16‐62/F	Sept	No	Yes (unknown date)	GD1a: 68	Ophthalmoplegia, No Limb weakness, Mild Dysphagia, Mild Dysarthria, No Respiratory Difficulty	No	IVIG
17‐38/F	Dec	No	No	GD1a: 84	Ophthalmoplegia, No Limb weakness, Mild Dysphagia, Mild Dysarthria, No Respiratory Difficulty Profound Lethargy, Positive MRI Brainstem Lesion Bickerstaff‐MFv Overlap	No	Plasma Exchange

Note

Summary of MFv cases seen at Adventist Health White Memorial (AHWM) and California Hospital Medical Center (CHMC) in 2015. Details of each case are detailed, including month of illness, presence of antecedent illness, recent vaccinations, ganglioside antibody panel, clinical features of illness, need for mechanical ventilation, and treatment received.

### 3.3 Ganglioside antibody profiles

Specific statistics are detailed in Table 3. Notable findings include that 50% of MFv patients tested positive for GQ1b; but none of these MFv patients tested positive for GM1 and GM2 antibodies. While the antibody panel for MFv patients is predominantly GQ1b, GBS patients were more evenly distributed; testing positive for all of the following: GD1a, GD1b, GQ1b, AsialoGM1, GM1, and GM2 (Table 3).

**Table 3:** Ganglioside antibody results

Antibody	GBS total	MFv total	Total
GD1a	3 (33.3%)	2 (25%)	5 (29.4%)
GD1b	5 (55.5%)	2 (25%)	7 (41.2%)
GQ1b	2 (22.2%)	4 (50%)	6 (35.3%)
AsialoGM1	2 (22.2%)	1 (12.5%)	3 (17.6%)
GM1	4 (44.4%)	0	4 (23.5%)
GM2	2 (22.2%)	0	2 (11.8%)

Note

Summary of ganglioside antibody panel for 17 patients diagnosed with GBS and MFv in Adventist Health White Memorial (AHWM) and California Hospital Medical Center (CHMC) in 2015.

### 3.4 Electrodiagnostic studies

Results of the nerve conduction tests are also detailed in Table 4. Of the 17 documented patients with GBS and MFv, five of the patients had undergone nerve conduction studies (NCS). All tested patients exhibited absent H waves and abnormal F waves. All the classic GBS patients were of the demyelinating subtype—half of whom had spared sural sensory nerve action potential (SNAP), and one‐quarter of whom had motor block present. The MFv patient that underwent NCS showed normal motor conduction with spared sural SNAP and no motor block.

**Table 4:** Nerve conduction study results for GBS and MFv cases

Patient	Diagnosis	Subtype	Sural SNAP	Motor block
3	Classic GBS	Demyelinating	Abnormal	None
4	Classic GBS	Demyelinating	Abnormal	None
6	Classic GBS	Demyelinating	Spared	Present
9	Classic GBS	Demyelinating	Spared	None
13	MFv	Normal motor conduction	Spared	None

Note

Summary of nerve conduction study (NCS) results for several documented cases of classic Guillain‐Barré Syndrome (GBS) and Miller Fisher variant (MFv) cases seen at Adventist Health White Memorial (AHWM) and California Hospital Medical Center (CHMC) in 2015. All cases detailed below demonstrated absent H waves and abnormal F waves. SNAP below refers to sensory nerve action potential.

## 4 DISCUSSION

Guillain‐Barré syndrome is a group of immune‐mediated neuropathies, and MFv makes up a small portion of its presenting variants. As previously mentioned, GBS has an incidence of 1‐2 cases per 100 000 person‐years worldwide.^3^ Of these GBS cases, MFv comprises 5% of cases in the United States and up to 25% of cases in Japan.^1^, ^6^ In the years prior to 2015, two large community hospitals in Downtown Los Angeles only saw approximately five cases of GBS per year, in 2016, seven GBS cases, two of which were MFv (29%), in 2017, eight GBS cases, two of which were MFv (25%), and in 2018, six GBS cases, one of which was MFv (17%). However, in 2015, there was greater than a threefold increase in GBS cases compared to years prior to 2015—with an unusually high number of MFv cases. Possible reasons for this dramatic increase in GBS cases may be an associated spike in gastrointestinal or upper respiratory infections, community stressors or natural disasters. However, no increases in associated infections were noted in the two community hospitals in 2015. Surges in GBS cases have been noted after large earthquakes in Japan, regional differences in exposure to infections, noninfectious stressors (eg, war), and H1N1 influenza vaccination.^3^, ^7^, ^12^, ^13^ There is a 14% increased risk of GBS in the winter compared to the summer.^14^ Similarly, in our patients, 75% of our documented GBS cases occurred in winter and spring. The seasonality of GBS also offers a clue to the common triggers of the disease, with upper respiratory infections being more common in the winter months.^14^

In addition to the high number of GBS cases in the DTLA areas in 2015, it is also notable that a high proportion of the GBS cases were Miller Fisher variants (47%). In comparison, the percentage of MFv in the United States is 5% and is reportedly over 25% in Japan.^6^ This markedly high percentage of MFv cases in our patient population may be because MFv is otherwise underdiagnosed when symptoms are mild. All of our MFv patients were unable to recall a prodromal illness (Table 2). Interestingly, each specific antecedent illness is associated with different presentation of MFv. For example, H. influenza infections are associated with the presenting symptom of diplopia and more often result in “pure MFv”—triad of ophthalmoplegia, ataxia, and areflexia without any limb weakness,^15^ whereas *C jejuni* infections are associated with blurred vision without diplopia and “incomplete MFv.” Cytomegalovirus (CMV) infections are associated with more severe disability with half of cases overlapping with GBS and Bickerstaff encephalitis.^15^ Due to the anti‐GQ1b antibody positivity, MFv is often presumed to be a homogenous disease entity; however, it is clear that there is variability in its presentation depending on the antecedent illness.

Gangliosides contain oligosaccharide head groups with multiple sialic acid residues. These lipids are found in high concentrations in ganglion cells and nerve endings. Gangliosides play many important roles in the proper function of nerve cells including cell adhesion, signal transduction, and receptor function.^10^ Each pathogenic microorganism is associated with a different set of ganglioside antibodies. This antibody patten is dependent on which ganglioside is most biochemically similar to the pathogen's lipopolysaccharides. Notably, GQ1b is associated with *C jejuni* infections and over 90% of MFv cases test positive for GQ1b IgG antibodies.^10^ Given the symptoms of ophthalmoplegia in MFv, it is no surprise that GQ1b ganglioside is found in high levels in the oculomotor, trochlear, and abducens nerves.^16^ In our MFv population, 50% of patient tested positive for GQ1b antibodies—however, antibody titers typically decrease rapidly with clinical improvement.^10^ It is possible that ganglioside antibody testing may have been delayed in our population, resulting in a lower percentage of GQ1b antibody positivity.

Guillain‐Barré syndrome symptoms typically occur 8‐10 days after antecedent illness, and nadir of symptoms occurs within 6 days of initial presentation. Symptoms improve and mostly self‐resolve in 1‐2 months.^5^ However, treating with IVIG and plasma exchange has been shown to decrease the median time until motor recovery and duration of mechanical ventilation.^5^, ^11^ Other proposed treatment modalities include eculizumab, eye patching, or prism therapy.^5^ Plasma exchange works by removing the cross‐reacting antibodies from circulation.^17^ Several proposed mechanisms of IVIG include inhibition of complement pathway, direct effects of remyelination, T‐cell modulation, and anti‐idiotype antibody production.^17^

## 5 CONCLUSION

Guillain‐Barré syndrome is a group of polyneuropathies that typically occurs after an antecedent illness that triggers the production of cross‐reacting antibodies that interfere with nerve function. Miller Fisher variant (MFv) is a subtype of GBS that presents with the classic triad of ophthalmoplegia, ataxia, and areflexia. The number of cases of GBS and MFv spiked threefold in downtown Los Angeles (DTLA) in 2015, with an unusually high proportion of MFv cases. Given this finding, it is plausible the MFv is regularly underdiagnosed when the accompanying neurological symptoms are mild. Further investigation into the health and community events of DTLA during the 2015 winter and spring season may yield more insight into the triggers of this disease. Additionally, further research into surges of other immune‐mediated neuropathies occurring in 2015 in the DTLA area may also provide more clues to their mechanism and treatment. Previously documented triggers include gastrointestinal and upper respiratory infections, natural disasters, and psychological stressors (eg, war). Overall, these findings emphasize the seasonality of GBS and its variants and offer more data regarding the diseases’ relation to ganglioside antibodies. This paper offers clear documentation of the GBS and MFv cases that occurred in the DTLA area in 2015—as well as an unusual predilection for the Miller Fisher variant.

## CONFLICT OF INTEREST

The authors declare that they have no competing interests.

## AUTHOR CONTRIBUTIONS

MY: drafted manuscript, analyzed data, and performed literature review of related topics. AL: had significant contributions in acquisition of data, revision of manuscript, and direct patient care involved in case. All authors read and approved the final manuscript.
